# Effects of types of air polishing powders on roughness, microhardness, color, and gloss of gingiva-colored resin-based composites

**DOI:** 10.1007/s00784-025-06404-3

**Published:** 2025-06-02

**Authors:** Özlem Saraç Atagün, Melek Almıla Erdoğan, Ülkü Tuğba Kalyoncuoğlu

**Affiliations:** 1 Department of Periodontology, Gülhane Faculty of Dentistry, University of Health Sciences, Ankara, Turkey; 2 Department of Prosthodontics, Gülhane Faculty of Dentistry, University of Health Sciences, Keçiören, Ankara, 06018 Turkey

**Keywords:** Air-polishing, Gingiva-colored composites, Color, Surface roughness

## Abstract

**Background:**

This study aimed to evaluate the effects of air polishing powders on the roughness, microhardness, color, and gloss of two types of gingiva-colored resin-based composites (GCRBCs).

**Methods:**

Disc-shaped specimens were prepared from two GCRBCs (Gradia Plus Gum, GC: Group G; Crea.lign, Bredent: Group C). Specimens subjected to air polishing were divided into two subgroups (*n* = 16): erythritol (Group G-e, Group C-e) and sodium bicarbonate (Group G-s, Group C-s). The roughness (Ra), microhardness (VHN), color (ΔE*_ab_ and Δ *E00*), and gloss (GU) values of all samples were measured before and after the applications. The data were analyzed by dependent *t*-test and two-way ANOVA.

**Results:**

Surface roughness showed statistically significant differences between initial and final measurements across all groups (G-s: *p* < 0.001; C-s: *p* = 0.002; G-e: *p* = 0.003; C-e: *p* = 0.011), with final Ra values being consistently higher than initial ones. Group C-e did not show significant differences (*p* = 0.294) in terms of microhardness after treatment, while hardness decreased in all other groups. In the final measurements, significant differences in color values were observed based on the type of powder applied (*p* = 0.026 for ΔE_ab_; *p* = 0.048 for ΔE_00_), with sodium bicarbonate causing more pronounced changes compared to erythritol. Significant differences were observed in initial and final gloss values for all subgroups (*p* = 0.00).

**Conclusion:**

It was observed that air polishing, particularly with sodium bicarbonate, can lead to significant roughness and discoloration in GCRBCs. Consequently, it is crucial to employ air-polishing devices correctly, limit their use on resin-based composite restorations, and opt for less abrasive polishing powders. These practices are essential to prevent increased surface roughness and surface microhardness and preserve color and gloss in GCRBCs.

## Introduction

When performing surgical or reconstructive procedures in the presence of alveolar resorption, resection, and asymmetry, soft and hard tissue augmentation may not always be feasible, requiring gingiva-colored materials to layer the framework materials [1]. Autopolymerizing acrylic resins, silicone materials, gingiva-colored porcelains, and composite resins can be used as gingiva-colored materials [2]. The most commonly used gingiva-colored materials are porcelains and indirect composite resins [1, 2]. Gingiva-colored porcelains are used to meet aesthetic demands in the gingival area; however, they have some disadvantages, such as the challenge of matching ceramics with soft tissues, the need for additional laboratory steps, distortion and shrinkage during multiple firings, and the complexity of repair procedures [2, 3]. Due to their low polymerization shrinkage, strong adhesion and shear strength to implant-supported frameworks, as well as their capacity for aesthetic characterization, gingiva-colored resin-based composites (GCRBCs) have emerged as a preferred alternative to porcelain for permanent restorations [2, 3, 4, 5].

Various instruments and techniques are available and scientifically proven for periodontal treatment, including hand instruments, ultrasonic scalers, and polishing devices [6]. The smoothing and polishing of the tooth surface is known as polishing in modern dental practice. This can be done manually or with motor-driven tools like strips, rubber prophylactic cups, nylon bristle brushes, air polishers, or vector systems in conjunction with a variety of prophylactic pastes and powders [7]. The procedure by which air-polishing systems work is that water and powder media are mixed in compressed air and applied to the tooth surface [8].

Surface roughness, which is typically assessed using the two-dimensional (2D) amplitude attribute Ra plays a critical role in plaque retention capacity [9]. It is well acknowledged that a surface roughness of 0.2 μm is the threshold for plaque retention [10]. According to the literature, reducing the surface roughness of dental composite resins also increases the microhardness and color stability, while potentially enhancing their gloss [11, 12]. It has also been reported that these mechanical and optical properties are affected by the chemical composition of the resin matrix (filler particle type, filler size, filler percentage, and filler-matrix bonding, etc.) as well as by environmental conditions and the polymerization methods [13]. The application of surface sealant agents to composite resin restorations aims to fill surface defects and reduce surface roughness. However, their long-term effectiveness has not been fully established [14].

Several studies have examined the effects of various air-abrasive powders on different dental materials during the maintenance phase of periodontal treatment [15, 16, 17, 18]. Moreover, there are some studies investigating the surface roughness [16, 17] and optical properties [11] of tooth-colored composite resins after air-polishing, however there are no studies investigating the microhardness. A recent study investigated how air-polishing affects the mechanical properties of a single type GCRBC with respect to power, angulation, duration, and powder type [19]. However, a comprehensive understanding of the roughness, microhardness, and optical properties of different GCRBCs after air-polishing with various powders is still needed. The present study aimed to evaluate the surface roughness, microhardness, color, and gloss of two different GCRBCs after air-polishing protocols using two different air-abrasive powders: erythritol and sodium bicarbonate.

The null hypothesis was that the use of different air-abrasive powders would not lead to significant differences in surface roughness, microhardness, color stability, and gloss among the tested groups.

## Materials and methods

### Sample preparation

**Fig. 1:**
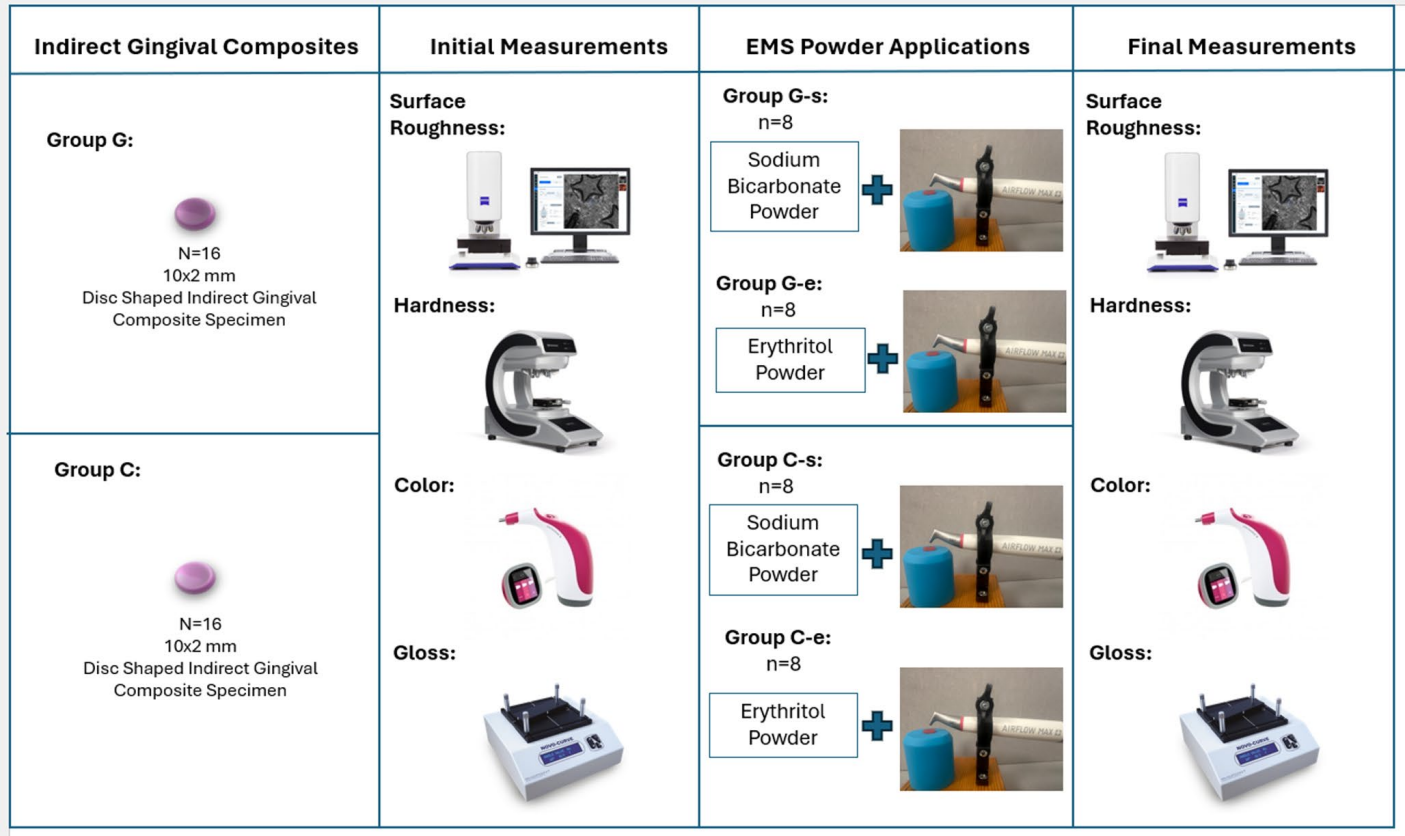
The flow chart of the study

The flow chart of the study is given in Fig. 1.

Two different gingiva-colored composites (Gradia Plus Gum, GC Europe NV, Leuven, Belgium, and Crea.lign Gum, Bredent GmbH, Senden Germany) materials were used. The brands and characteristics of the materials examined are shown in Table 1.

**Table 1:** Materials used in this study

Material	Manufacturer	Composition	LOT #
Gradia Gum (GRG)	GC Europe NV Leuven, Belgium	Resin: UDMA, NGDMA, TMPTMA Filler: Trimodal (pre-polymerized particles, AlBSiO_4_, SiO_2_, 75 wt%)	2,305,011
Crea.lign GUM (crea.lign)	Bredent, Senden, Germany	Resin: BisGMA, UDMA and aliphaticdimethacrylate resins Filler: Inorganic nano-ceramic filler (50%)	N231752
Erythritol	EMS, Nyon, Switzerland	Erythritol 14 μm, Cetyl Pyridinium Chloride 0.05%	2,311,072
Sodium Bicarbonate	EMS, Nyon, Switzerland	Sodiumhydrogencarbonate 40 μm, hydrophobe modified silica, lemon taste	FB-143
Optiglaze	GC Corporation, Tokyo, Japan	PMMA, MMA, silica filler, photo inhibitor	2,303,201

The main hypothesis of the research was to investigate the differences between independent groups. Similar studies that can be used in sample size calculation were examined, and the sample size calculation that gives the highest number according to the statistical methods to be applied in line with the main hypotheses was taken into consideration. In this study, using the ‘G. Power-3.1.9.2’ program [20], at a 95% confidence level (α = 0.05), the standardized effect size was calculated as 0.6437 from a similar study [2] (Table 2, UV aging), and the minimum sample size for each group was obtained as 8 with a theoretical power of 0.80.

**Table 2:** Distribution and comparison of microhardness measurements according to material, applications, and measurement time

		Initial			Final			Time-Dependent		
Groups		Min -Max.		Mean ± S.D	Min -Max.		Mean ± S.D	Test Statistic		*P*
G-s		42.7–70		60.97 ± 9.55(62.35)	25.3–58.2		40.34 ± 11.54(38.08)	6.43		< 0.001*
C-s		26-31.1		28.42 ± 1.65(28.2)	12.71-24		17.16 ± 4.25(16.65)	7.06		0.001*
G-e		44.4–68.2		58.15 ± 8.99(56.95)	19.7-56.41		40.72 ± 13.47(41.92)	3.61		0.015*
C-e		26.3–36		31.17 ± 3.66(30.3)	23.9–35.5		28.87 ± 4.28(27.55)	1.17		0.294
After Application			Type 3 Sum of Squares		Sd	Sum of Squares			F	*P*
Material Application			0.41		1	0.41			5.62	0.028*
Material			0.11		1	0.11			1.53	0.229
Material*Material Application			0.23		1	0.23			3.26	0.086

*p < 0.05

32-disc-shaped specimens with thicknesses of 2 mm and diameters of 10 mm were prepared. A plastic mold was filled with indirect composite resin. A cellulose strip band and a glass slide were placed in the mold, applying finger pressure. Both surfaces of the indirect composite resins were pre-polymerized for 40 s with an LED light device (DTE LUX-E Plus, Guilin Woodpecker Medical Instrument, Guilin, Guangxi, China; 1200 mW/cm²) followed by an additional 3 min of polymerization in the Labolight DUO polymerization device (GC Europe NV, Leuven, Belgium).The grinding was carried out with the help of a bench-top laboratory polishing grinder (Forcipol 202 and Forcimat 52, Metcon Insturements, Osmangazi, Bursa, Turkey) using silicon carbide sandpapers (500 to 1200 grits) (Atlas, Saint-Gobain Abrasives, Istanbul, Turkey).

Polishing was performed with diamond-impregnated polishing discs (Diacomp Plus Twist Set RA 342, EVE Technik, Pforzheim, Germany) at a speed of 10,000 rpm for 20 s. Subsequently, the samples were cleaned in an ultrasonic cleaner with distilled water for 5 min and air-dried. Any surface irregularities of the indirect composite were smoothed out, and the surface was cleaned thoroughly. Following the manufacturer’s instructions, Optiglaze Color Clear (GC Europe NV, Leuven, Belgium) was applied as a surface coating agent using a clean brush [21]. Finally, the samples were polymerized in the Labolight DUO polymerization device for 90 s.

The prepared GCRBCs were classified into Group G (Gradia Plus Gum, GC) and Group C (Crea.lign, Bredent), and further subdivided into subgroups as Group G-E, Group C-E (erythritol); Group G-S, and Group C-S (sodium bicarbonate) based on the types of powders they were exposed to.

Erythritol and sodium bicarbonate powders were applied to the Optiglaze-coated surfaces of the specimens using the EMS Airflow^®^ Prophylaxis Master. (EMS, Nyon, Switzerland). Parts of an adjustable, customized jig were 3D printed using the Creality Ender V3 printer (Creality 3D Technology Co., Shenzhen, China) using the stereolithography technique. Polylactic acid (PLA) resin (FilameX PLA, İstanbul, Turkey) was used to print the pieces, which were then put together with steel M3 bolts and nuts and secured to a wooden block. Throughout the procedure, the samples were maintained stationary and fixed on a horizontal platform. The powder applications were conducted at a 5 mm distance from the target surface and a 45-degree angle, ensuring effective treatment and even distribution of the material.

The process utilized full power settings (air pressure: 700 kPa; water pressure: 500 kPa) to maximize the efficiency of the application, while a continuous flow of water was incorporated to cool the area and enhance the effectiveness of the treatment. Each application lasted for 10 s [22], allowing sufficient time for the material to interact with the surface without causing undue stress or damage. This carefully controlled methodology was designed to ensure reproducibility and accuracy in the assessment of the results.

### Surface roughness (Ra)

Surface roughness measurements before and after erythritol or sodium bicarbonate applications were made using a 3D optical profilometer (Zeiss Smartproof 5, Carl Zeiss, Jena, Germany). From each specimen center point, in 3D imaging mode, at a total magnification of 20x (C Epiphalan-Apochromat 20x/0.7 DIC, Carl Zeiss, 39Jena, Germany), a 500 μm x 500 μm area was scanned in fast mode (4 μm) and 3 random readings were performed without a filter. The images obtained were transferred to the automated software analysis program (ConfoMap ST 7.4.8076, Carl Zeiss, Jena, Germany). The arithmetic averages of the Ra values ​​taken 3 times were recorded.

### Microhardness (HVkp)

Microhardness measurements before and after erythritol or sodium bicarbonate applications were performed by a hardness tester (Shimadzu HMV-G Micro Hardness Tester, Japan) equipped with a Vickers indenter under a 0.98-N load with a 10-s contact period and 70x magnification. On each specimen, three indentations were made in an equilateral triangular mode 2 mm distant from the margins, and the HVkp was averaged.

### Color measurements

CIELAB color space (CIE: International Commission on Illumination) was used to determine the color differences [23]. The primary parameters of color (L* lightness from black (0) to white (100), a* amount of green (-) and red (+), b* amount of blue (-), and yellow (+)) of all specimens in the CIELAB (ΔE*ab) total color differences were recorded before and after air-polishing procedures with a spectrophotometer on white baseline (L* = 98.2, a* = −0.14, b* = −0.24) (Vita Easyshade V, VITA Zahnfabrik, Germany). The colorimeter was calibrated at the beginning and then after every 20 measurements as per the manufacturer’s instructions. Color measurements were made in a color measurement cabin to ensure standardization. The inside of the color measurement cabin was covered with a gray floor,

and the. Measurements were made with three measurements from the center point of each specimen. Color differences (ΔE*ab and Δ *E*_00_) were calculated based on the formulas below [24]. CIELAB (Δ E*ab) formula was used as follows

$$\:{\varDelta\:E}^{*}ab=\sqrt{{\left({\varDelta\:L}^{*}\right)}^{2}+{\left({\varDelta\:a}^{*}\right)}^{2}+{\left({\varDelta\:b}^{*}\right)}^{2}}$$

CIEDE2000 (Δ E_00_) formula was used as follows:

$$\begin{array}{l}\:{\varDelta\:E}_{00}\\=\sqrt{{\left(\frac{{\varDelta\:L}^{{\prime\:}}}{{K}_{L}{S}_{L}}\right)}^{2}+{\left(\frac{{\varDelta\:C}^{{\prime\:}}}{{K}_{C}{S}_{C}}\right)}^{2}+{\left(\frac{{\varDelta\:H}^{{\prime\:}}}{{K}_{H}{S}_{H}}\right)}^{2}+{R}_{T}\left(\frac{{\varDelta\:C}^{{\prime\:}}}{{K}_{C}{S}_{C}}\right)\left(\frac{{\varDelta\:H}^{{\prime\:}}}{{K}_{H}{S}_{H}}\right)}\end{array}$$

The results and dimensions of a reference study are used to evaluate color variations in GCRBCs [25] This scale indicated that the perceptual threshold limit was reached when △E*ab ≤ 1.7 and ΔE_00_ ≤ 1.1, which was regarded as an excellent match.

### Gloss

The Gloss, (Gs in GU) of the before and after erythritol or sodium bicarbonate applications of each specimen was measured with a glossmeter (Novo-Cure, Rhopoint Instruments Ltd, East Sussex, UK) at a 60°-angle through a 4.5-mm diameter aperture. Manufacturer’s calibration tile was used before the measurement. The specimens were positioned over the glossmeter with the central section oriented to the glossmeter aperture, and an opaque device covered the sample in order to obstruct ambient light exposure during the measurement. Three measurements of Gs were taken from each sample. The same area was assessed, but the sample was rotated roughly 33.3° between measurements to acquire Gs values from various angles. The mean of these measurements was regarded as the sample’s Gs.

### Statistical analysis

This study gives descriptive statistics of the data (mean, standard deviation, median, minimum, and maximum). The assumption of normal distribution was checked by Shapiro Wilk test, homogeneity of variance by Levene’s test, and sphericity assumption by Mauchly’s W test. In cases where the normality assumption was met, the Dependent Sample T-test was used to compare two dependent groups. The two-way ANOVA test was used to examine the difference between independent groups where the normality assumption was met with the interaction effect. Analyses were performed in the IBM SPSS 25 program.

## Results

**Fig. 2:**
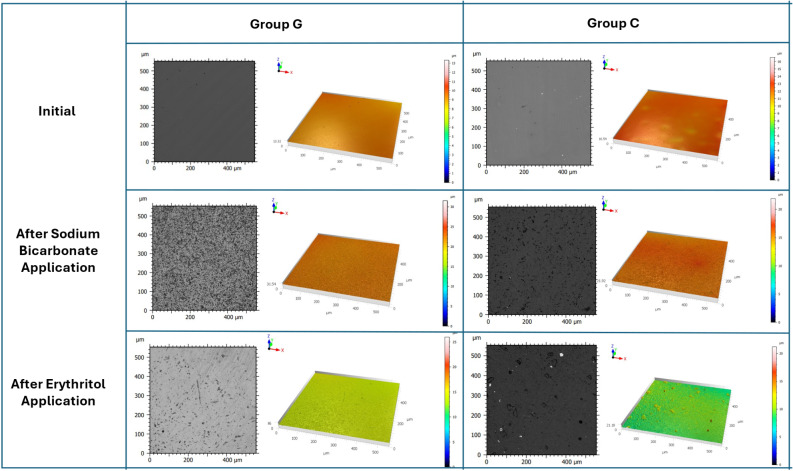
Visualization of the surface roughness profile of indirect composites before and after treatment

The appearance of the surface roughness profile of the indirect composites before and after processing is presented in Fig. 2.

The Table 3 shows the roughness measurements’ distribution and comparison based on the applied powder, materials, and measurement time.

**Table 3:** Distribution and comparison of roughness measurements according to material, applications, and measurement time

		Initial Ra			Final Ra		Time-Dependent	
Groups		Min.-Max.		Mean ± S.D	Min.-Max.	Mean ± S.D	Test Statistic	*P*
G-s		0.01–0.04		0.02 ± 0.01(0.02)	0.22–0.27	0.24 ± 0.02(0.24)	-22.46	< 0.001*
C-s		0.02–0.05		0.04 ± 0.01(0.04)	0.15–0.31	0.23 ± 0.06(0.21)	-8.02	0.002*
G-e		0.02–0.08		0.04 ± 0.02(0.04)	0.09–0.18	0.13 ± 0.04(0.13)	-5.78	0.003*
C-e		0.02–0.09		0.05 ± 0.02(0.05)	0.1–0.2	0.14 ± 0.04(0.13)	-4.05	0.011*
After Application			Type 3 Sum of Squares		Sd	Sum of Squares	F	*P*
Material Application			0.06		1	0.06	34.38	< 0.001*
Material			0.00		1	0.00	0.07	0.784
Material*Material Application			0.00		1	0.00	0.41	0.529

*p < 0.05

As a result of the analysis for measurement times, statistically significant differences were determined between Ra measurements according to time in all materials and applications (*p* < 0.05). The final measurements were higher than the initial measurements. As a result of the analysis in the final measurements, statistically significant differences were found between Ra measurements according to applications, but no statistically significant differences were found between Ra measurements according to materials, and the material*application interaction effect was found to be insignificant (*p* > 0.05).

The distribution and comparison of hardness measurements according to material, applications, and measurement time are given in Table 2.

As a result of the analysis for measurement times, statistically significant differences were found between hardness measurements according to time for Group G-s, Group C-s, and Group G-e (*p* < 0.05). The initial measurements were higher than the final measurements. For the analyses performed for Group C-e, no statistically significant difference was obtained between hardness measurements according to time (*p* > 0.05). In the final measurements, statistically significant differences were found between the hardness measurements according to the applied powder (*p* < 0.05). Still, statistically significant differences were not obtained between the hardness measurements according to the material, and the material*application interaction effect was found to be insignificant (*p* > 0.05).

**Fig. 3:**
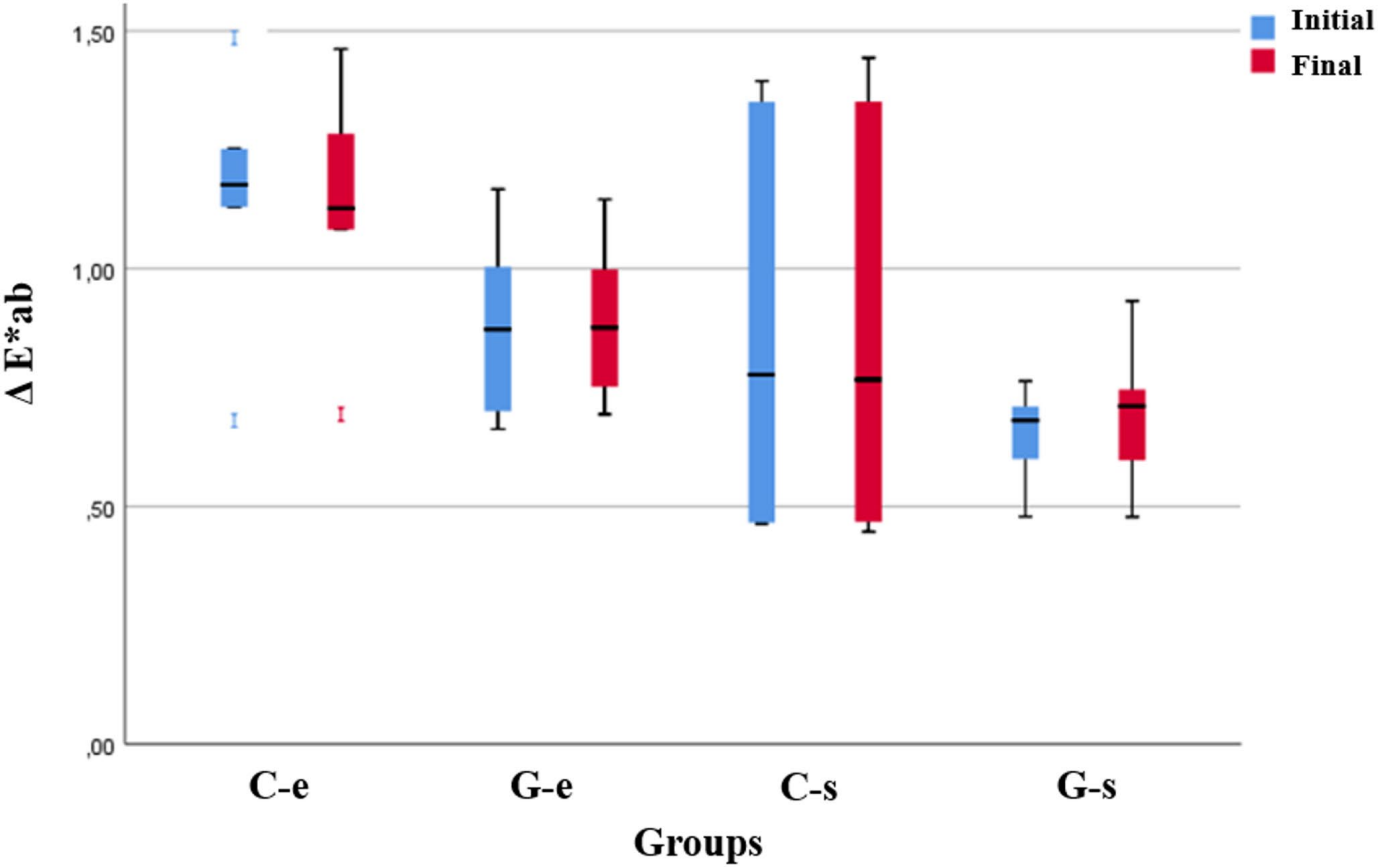
Box plots of the distribution of color measurements by applications and measurement time

Table 4 shows the distribution and comparability of color measurements by material, application, and measurement period. As a result of the analysis for measurement times, no statistically significant differences were obtained between ΔE measurements according to time in all materials and applications (*p* > 0.05). In the final measurements, statistically significant differences were found between the color measurements according to the powder applied (*p* < 0.05) (Fig. 3), statistically significant differences could not be obtained between the color measurements according to the materials, and the material*application interaction effect was found to be insignificant (*p* > 0.05).

**Table 4:** Distribution and comparison of color change measurements according to material, applications, and measurement time

			ΔE_ab_				ΔE_00_			
Groups			Min.-Max.	Mean ± S.D			Min.-Max.	Mean ± S.D		
G-s			0.48–0.76	0.65 ± 0.11(0.68)			0.48–0.93	0.69 ± 0.15(0.71)		
C-s			0.46–1.39	0.87 ± 0.41(0.78)			0.45–1.44	0.87 ± 0.43(0.77)		
G-e			0.66–1.17	0.88 ± 0.2(0.87)			0.69–1.15	0.89 ± 0.18(0.88)		
C-e			0.68–1.49	1.15 ± 0.26(1.18)			0.69–1.46	1.13 ± 0.26(1.13)		
ΔE_ab_		Type 3 Sum of Squares			Sd	Sum of Squares			F	*P*
Material Application		0.40			1	0.40			5.73	0.026*
Material		0.37			1	0.37			5.39	0.030*
Material*Material Application		0.00			1	0.00			0.05	0.823
ΔE_00_		Type 3 Sum of Squares			Sd	Sum of Squares			F	*P*
Material Application		0.32			1	0.32			4.42	0.048*
Material		0.28			1	0.28			3.81	0.064
Material*Material Application		0.00			1	0.00			0.05	0.814

*p < 0.05

Table 5 shows how gloss measurements are distributed and compared by material, application, and measurement period.

**Table 5:** Distribution and comparison of gloss measurements according to material, applications, and measurement time

		Initial			Final				Time-Dependent		
Groups		Min.-Max.		Mean ± S.D	Min.-Max.			Mean ± S.D	Test Statistic		*P*
G-s		72.1–90.8		82.22 ± 6.58(82.65)	16.8–50.2			30.43 ± 12.51(29.85)	15.93		< 0.001*
C-s		70.1–88.6		80.82 ± 6.58(81.8)	10.4–29.4			20.08 ± 8.75(21.05)	22.87		< 0.001*
G-e		72.9–91.1		83.62 ± 6.17(85.2)	38.4–68.6			53.03 ± 11.38(55.8)	12.12		< 0.001*
C-e		71.7–90.7		81.63 ± 8.03(82.6)	45.6–68.3			55.47 ± 8.48(56.4)	6.96		0.001*
After Application			Type 3 Sum of Squares			Sd	Sum of Squares			F	*P*
Material Application			5043.10			1	5043.10			46.41	< 0.001*
Material			94.01			1	94.01			0.86	0.363
Material*Material Application			245.12			1	245.12			2.25	0.149

*p < 0.05

**Fig. 4:**
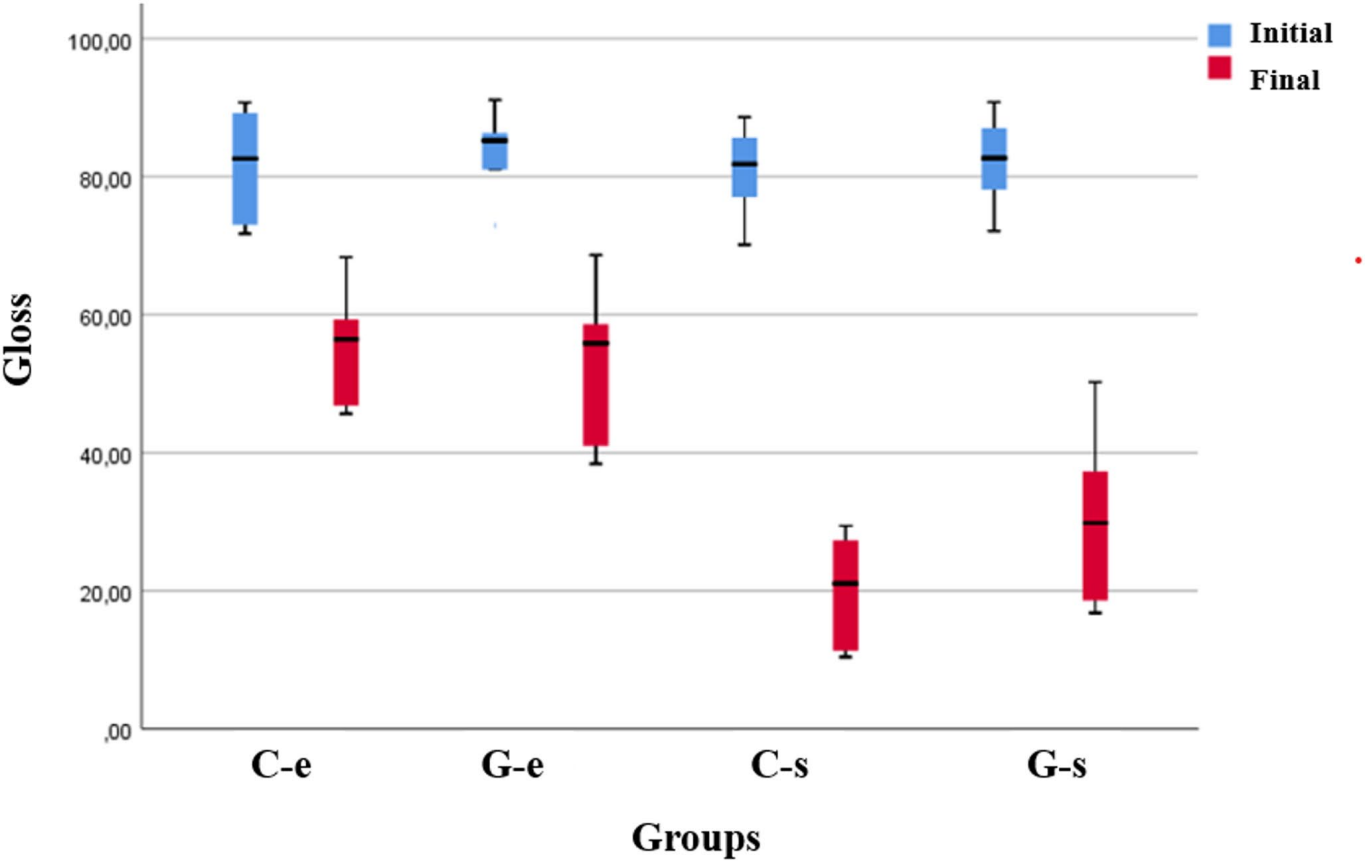
Box plots of the distribution of gloss measurements by application and measurement time

As a result of the analysis for measurement times, statistically significant differences were determined between gloss measurements according to time for all materials and applications (*p* < 0.05). The initial measurements were higher than the final measurements. A statistically significant difference was found between the gloss measurements according to the powder applied in the measurements made after the applications (Fig. 4).

Still, no statistically significant differences were obtained between the gloss measurements according to the materials, and the material*application interaction effect was determined to be insignificant (*p* > 0.05).

## Discussion

This in vitro study provides comprehensive information on the effects of two types of commercially available and commonly used air polishing powders, based on sodium bicarbonate and erythritol, on the surface roughness, hardness, color, and gloss of GCRBCs.

Based on the findings of this study, the null hypothesis, which stated that different air-abrasive powders would not create significant differences in surface roughness, microhardness, color stability, or gloss among various GCRBCs, was partially accepted.

Different composite materials may have distinct resin compositions as well as filler particles with varying sizes and compositions. The sensitivity of the surface to damage may therefore vary [17]. For example, although the better polishability and aesthetic appearance of nanofill resin-based composites are considered superior to nano- or micro-hybrid resin-based composites, due to the larger surface area/volume ratio of the filler particle system, nanofill resin-based composites may undergo a higher degree of degradation during function in the oral cavity [26]. Nemeth et al. reported that the effect of air polishing on micro-hybrid resin-based composite was more than three times, while it was less aggressive on nano-filler resin-based composite [27]. Crea-lign, which contains only nanoparticles and no glass filler, and Gradia, which contains prepolymer and glass particles as filler, showed similar mechanical and optical properties after air-polishing. However, the fact that C-e is the only group that does not show a significant difference in hardness measurements depending on time can be explained by the higher durability of Crea-lign due to its filler content.

Periodontitis is a major cause of tooth loss in adults [28]. As a result, patients with periodontal disease are more likely to have fixed or removable prosthetic restorations compared to periodontally healthy individuals [29]. This highlights the clinical relevance of evaluating how decontamination procedures affect restorative materials in such patients. Polishing is routinely performed during periodontal treatment, both to remove the biofilm layer and to create a brighter and smoother surface after the initial periodontal treatment [30]. The particle size, hardness, and angularity of the abrasive powder all affect the surface damage caused by kinetic abrasion [17]. The use of air-polishing with Erythritol and Glycine powders in non-surgical periodontal or peri-implant prophylaxis and treatment has been reported to be effective, comfortable, and safe on natural teeth and implants [31]. However, it should not be forgotten that many of these patients also have prosthetic restorations. Given their particular uses near or in touch with gingival tissues, this is even more crucial for gingiva-colored composites [3]. Turunç et al. reported that various air-polishing powders negatively affected the color stability of different CAD/CAM restorative materials [18]. Babina et al. reported that air-polishing with calcium carbonate and sodium bicarbonate powders caused a greater increase in the surface roughness of composite resins and restorations [32]. Gomes et al. reported that the prophylaxis technique that uses the bicarbonate jet afforded a significant increase in the surface roughness of resin composite restorations [22]. Demirci et al. reported that the air-polishing process increased the surface roughness of ceramic-filled polyetheretherketone (PEEK) composites [33]. We similarly observed that air-polishing increased the surface roughness of GCRBCs, and these effects were more pronounced when sodium bicarbonate powder was used. Thus first null hypothesis was rejected.

Given its connection to abrasion resistance, hardness is a crucial characteristic of composite materials. From a therapeutic perspective, a higher hardness is generally preferred since it increases resistance to wear and scratches and, consequently, the ability to retain the original surface characteristics (i.e., gloss, roughness) [3]. It is known that increased water absorption leads to a decrease in the hardness of resins [34]. In this study, significant decreases occurred in hardness values after application in all groups except the C-e group. This can be explained by the lower surface abrasive effect of erythritol compared to sodium bicarbonate and the higher microhardness of group C compared to group G.

For patients, the aesthetic appearance of the restorative materials is highly significant, and color stability is essential for a smile that looks good [18]. GCRBCs must be color stable to preserve their natural and beautiful appearance throughout time since any color alterations or discolorations could jeopardize the effectiveness of treatment and patient satisfaction [35]. Color stability is influenced by the kind and proportion of the monomer matrix, with more hydrophobic monomers being more stable [36]. Douglas et al. also demonstrated that hydrophobic monomers reduce water uptake and improve resistance to discoloration [37]. Benavides-Reyes et al. revealed that different GCRBCs have different color stability and are differentially affected by different coloring procedures [35]. In this study, it was observed that the color stability of two different GCRBCs decreased statistically insignificantly after air-polishing; the effect of sodium bicarbonate was higher than that of erythritol, but there was no significant difference in terms of the composites examined. Similarly, Kalyoncuoğlu et al. reported that the mean ΔE∗ab values of all GCRBCs after UV aging were at acceptable levels [2].

The amount of light reflected by a surface at the same angle as the incident light is known as the gloss value and is a metric used to examine how smooth a surface is. Although Chiang et al. found a significant correlation between surface gloss and subjective perception of surface texture, filler size, loading, and distribution also affect surface gloss [38]. Filler bonding has been proposed for composite resins to achieve smoother surfaces. In this study, the surfaces of composite resin samples were coated with Optiglaze according to the manufacturer’s instructions to mimic clinical practice. It was observed that the surface gloss decreased and the surface roughness increased after applying both types of air polishing powder. This might be attributed to the wear of the optiglaze layer on the sample surfaces. Furthermore, previous studies reported that acrylic resin samples coated with Optiglaze exhibit higher hardness values [39]. The decrease in micro-hardness values observed in the present study may be attributed to the abrasion of the Optiglaze layer caused by air-polishing powders. The viscous nature of Optiglaze, owing to its filler content, may have limited its ability to spread evenly over composite surfaces [40], making it more susceptible to mechanical removal. Moreover, variations in filler composition, resin matrix structure, and surface energy among different composites could affect how well the Optiglaze layer adheres and responds to mechanical stress, potentially influencing its resistance to abrasive procedures [41]. Some studies have suggested that Optiglaze may increase surface staining due to nanofiller detachment from the resin matrix, leading to surface voids [42, 43]. In contrast, other studies have shown that Optiglaze significantly enhances the color stability of composite resins compared to other surface sealants [14]. In the present study, despite the use of two different composite resins, no significant differences were observed between them in terms of surface roughness, gloss, or micro-hardness, suggesting a comparable performance of Optiglaze coating across these materials under air-polishing conditions. In clinical practice, reapplication of Optiglaze may be a practical approach to recover esthetic and mechanical properties after air-polishing-induced wear, but further in vitro studies are needed to assess the long-term durability and effectiveness of repeated coatings or polishing procedures.

This study has several limitations. First of all, the distance, contact pressure parameters, and application angle were kept constant in this study. In addition, both the holder made for this purpose and the platform with the treated sample were kept stationary throughout the experiment. Considering that the airflow head is in motion during the polishing process in the mouth, our experimental setup may not have accurately simulated reality. The in vitro nature of the study is another limitation in itself due to the different intraoral temperature and pH values and the lack of a washing effect of saliva. The results are further constrained by the fact that no plaque replacements were used in this investigation, as its goal was to test roughness, microhardness, color, and gloss rather than efficacy. The lack of a control group in which optiglaze was not applied can also be considered as a limitation. The amount of optiglaze and composite removed from the surface after air polishing should be evaluated by advanced techniques. - One additional limitation of this study is the lack of long-term simulation (e.g., thermocycling, aging) which may influence the durability of surface effects. Further in vitro and clinical studies incorporating additional parameters to better simulate air-polishing treatments are needed for a comprehensive evaluation.

## Conclusion

Sodium bicarbonate air polishing caused notable material loss in GCRBCs. Proper use of air-polishing devices—by limiting exposure time and selecting less abrasive powders—was essential to minimize surface roughness, preserve hardness, and maintain the aesthetic properties of resin-based composite restorations.

## Data Availability

No datasets were generated or analysed during the current study.

## References

[1] Lucena C, Benavides-Reyes C, Ruiz-López J, Tejada-Casado M, Pulgar R, Pérez MM (2023) Relevant optical properties for gingiva-colored resin-based composites. J Den 126:104316 10.1016/j.jdent.2022.104316 36195249

[2] Kalyoncuoğlu ÜT, Yilmaz B, Sipahi C (2023) Determination of the degree of conversion, the diffuse reflectance, and the color stability after different aging processes of gingiva-colored composite resins. J Prosthodont 32:743–751 37291715 10.1111/jopr.13721

[3] Petropoulou A, Dimitriadi M, Zinelis S, Sarafianou A, Eliades G (2020) Surface characteristics and color stability of gingiva-colored resin composites. Mater 13:2540 10.3390/ma13112540 PMC7321486 32503174

[4] An HS, Park JM, Park EJ (2011) Evaluation of shear bond strengths of gingiva-colored composite resin to porcelain, metal and zirconia. J Adv Prosthodont 3:166–171 22053249 10.4047/jap.2011.3.3.166 PMC3204454

[5] Fushiki R, Komine F, Kimura F, Kusuba K, Kondo T, Moriya Y et al (2019) Bond strengths between gingiva-colored layering resin composite and zirconia frameworks coated with feldspathic porcelain. Dent Mater J 38:547–554 31105163 10.4012/dmj.2018-253

[6] Bühler J, Amato M, Weiger R, Walter C (2016) A systematic review on the effects of air Polishing devices on oral tissues. Int J Dent Hyg 14:15–28 25690301 10.1111/idh.12120

[7] Chowdhary Z, Mohan R (2018) Efficiency of three different Polishing methods on enamel and cementum: A scanning electron microscope study. J Indian Soc Periodontol 22:18–24 29568167 10.4103/jisp.jisp_40_17 PMC5855262

[8] Petersilka P GJ (2011) Subgingival air-polishing in the treatment of periodontal biofilm infections. Periodontol 2000 55:124–142 21134232 10.1111/j.1600-0757.2010.00342.x

[9] Teranaka A, Tomiyama K, Ohashi K, Miyake K, Shimizu T, Hamada N, Mukai Y, Hirayama S, Nihei T (2018) Relevance of surface characteristics in the adhesiveness of polymicrobial biofilms to crown restoration materials. J Oral Sci 60:129–136 29162785 10.2334/josnusd.16-0758

[10] Bollen C, Lambrechts P, Quirynen M (1997) Comparison of surface roughness of oral hard materials to the threshold surface roughness for bacterial plaque retention: A review of the literature. Dent Mater 13:258–269 11696906 10.1016/s0109-5641(97)80038-3

[11] Vargas RP, Machado AC, da Silva GR, Miranda AS, Campolina MG, Santos-Filho PC, Menezes MS (2024) Influence of different finishing, aging with coffee, and repolishing protocols on the properties of nanoparticle composite resins. J Clin Exp Dent 16:e724–e32 39130361 10.4317/jced.61653 PMC11310979

[12] Jain V, Platt JA, Moore K, Spohr AM, Borges GA (2013) Color stability, gloss, and surface roughness of indirect composite resins. J Oral Sci 55:9–15 23485595 10.2334/josnusd.55.9

[13] Stawarczyk B, Egli R, Roos M, Ozcan M, Hämmerle CH (2011) The impact of in vitro aging on the mechanical and optical properties of indirect veneering composite resins. J Prosthet Dent 106:386–398 22133396 10.1016/S0022-3913(11)60153-4

[14] Şahin O, Dede DÖ, Köroğlu A, Yılmaz B (2015) Influence of surface sealant agents on the surface roughness and color stability of artificial teeth. J Prosthet Dent 114:130–137 25913372 10.1016/j.prosdent.2015.02.009

[15] Gunawan V, Carrington SD, Choi YJ, Choi JJE (2024) Air-polishing technology is an effective alternative chairside method for cleaning dentures. Int J Dent Hyg 22:626–638 37680139 10.1111/idh.12735

[16] Reinhart D, Singh-Hüsgen P, Zimmer S, Bizhang M (2022) In-vitro influence of the use of an erythritol powder through air Polishing on the surface roughness and abrasiveness of various restorative materials. PLoS ONE 17(7):e0270938 35797310 10.1371/journal.pone.0270938 PMC9262204

[17] Janiszewska-Olszowska J, Drozdzik A, Tandecka K, Grocholewicz K (2020) Effect of air-polishing on surface roughness of composite dental restorative material - comparison of three different air-polishing powders. BMC Oral Health 20:1–7 10.1186/s12903-020-1007-y PMC6993449 32000753

[18] Turunç Oğuzman R, Yüzbaşıoğlu E (2023) Air-polishing powders’ effect on the color of CAD/CAM restorative materials. Appl Sci 13:11573

[19] Atagün ÖS, Kalyoncuoğlu ÜT (2025) Influence of powder type, power, angulation and duration in air-polishing on the surface properties of gingiva-colored composites. BMC Oral Health 25(1):581 40241042 10.1186/s12903-025-05987-3 PMC12004825

[20] Faul F, Erdfelder E, Buchner A, Lang AG (2009) Statistical power analyses using G*Power 3.1: tests for correlation and regression analyses. Behav Res Methods 41:1149–1160 19897823 10.3758/BRM.41.4.1149

[21] Erdogan MA, Kalyoncuoğlu ÜT, Yilmaz Erdemli B (2025) Comparative effects of conventional and electronic cigarettes on discoloration and surface roughness of gingiva-colored dental materials. Journal of Prosthodontics 10.1111/jopr.14054 PMC12147406 40170561

[22] Gomes IA, Mendes HG, de Nina CRC, Turssi MG, CP Vasconcelos (2018) Effect of dental prophylaxis techniques on the surface roughness of resin composites. J Contemp Dent Pract 19:37–41 29358532 10.5005/jp-journals-10024-2208

[23] Grieco PC, Silva D, Ishida JD, Y., Ishikawa-Nagai S (2021) An in vivo spectrophotometric analysis of gingival acrylic shade guide. Materials 14(7):1768 33916651 10.3390/ma14071768 PMC8038330

[24] Ayaz EA, Altintas SH, Turgut S (2014) Effects of cigarette smoke and denture cleaners on the surface roughness and color stability of different denture teeth. J Prosthet Dent 112:241–248 24787128 10.1016/j.prosdent.2014.01.027

[25] Paravina RD, Pérez MM, Ghinea R (2019) Acceptable and perceptibility thresholds in dentistry: A comprehensive review of clinical and research applications. J Esthet Restor Dent 31:103–112 30891913 10.1111/jerd.12465

[26] Almeida GS, Poskus LT, Guimarães JGA, da Silva EM (2010) The effect of mouthrinses on salivary sorption, solubility and surface degradation of a nanofilled and a hybrid resin composite. Oper Dent 35:105–111 20166417 10.2341/09-080-L

[27] Németh KD, Haluszka D, Seress LV, Lovász BV, Szalma J, Lempel E (2022) Effect of air-polishing and different post-polishing methods on surface roughness of nanofill and microhybrid resin composites. Polymers 14:1643 35566812 10.3390/polym14091643 PMC9100913

[28] Darby I (2022) Risk factors for periodontitis & peri-implantitis. Periodontol 2000 90(1):9–12 35913624 10.1111/prd.12447 PMC9804916

[29] Curtis DA, Lin GH, Rajendran Y, Gessese T, Suryadevara J, Kapila YL (2021) Treatment planning considerations in the older adult with periodontal disease. Periodontol 2000 87(1):157–165 10.1111/prd.12383 34463978

[30] Cobb CM, Daubert DM, Davis K, Deming J, Flemmig TF, Pattison A, Stambaugh RV (2017) Consensus conference findings on supragingival and subgingival air polishing. Compend Contin Educ Dent 38 (2):e1–e4 28156118

[31] Liu CC, Dixit N, Hatz CR (2024) Air powder waterjet technology using erythritol or glycine powders in periodontal or peri-implant prophylaxis and therapy: A consensus report of an expert meeting. Clin Exp Dent Res 10:e855 38345462 10.1002/cre2.855 PMC10860664

[32] Babina K, Polyakova M, Sokhova I, Doroshina V, Arakelyan M, Zaytsev A, Novozhilova N (2021) The effect of ultrasonic scaling and air-powder Polishing on the roughness of the enamel, three different nanocomposites, and composite/enamel and composite/cementum interfaces. Nanomaterials (Basel) 11:3072 34835835 10.3390/nano11113072 PMC8623571

[33] Demirci F, Birgealp Erdem M, Tekin S, Caliskan C (2022) Effect of ultrasonic scaling and air Polishing on the surface roughness of polyetheretherketone (PEEK) materials. Am J Dent 35:200–204 35986936

[34] Gibreel M, Perea-Lowery L, Vallittu PK, Garoushi S, Lassila L (2022) Two-body wear and surface hardness of occlusal splint materials. Dent Mater J 41:916–922 36288940 10.4012/dmj.2022-100

[35] Benavides-Reyes C, Pérez MM, Tejada-Casado M, Ruiz-López J, Lucena C (2023) Color stability and degree of conversion of gingiva-colored resin-based composites. J Esthet Restor Dent 35:896–903 37403541 10.1111/jerd.13082

[36] Fonseca ASQS, Labruna Moreira AD, de Albuquerque PPAC, de Menezes LR, Pfeifer CS, Schneider LFJ (2017) Effect of monomer type on the CC degree of conversion, water sorption and solubility, and color stability of model dental composites. Dent Mater 33:394–401 28245929 10.1016/j.dental.2017.01.010

[37] Douglas WH, Craig RG, Chen CJ (1979) A new composite restorative based on a hydrophobic matrix. J Dent Res 58(10):1981–1986 291622 10.1177/00220345790580100401

[38] Chiang YC, Lai EHH, Kunzelmann KH (2016) Polishing mechanism of light-initiated dental composite: geometric optics approach. J Formos Med Assoc 115:1053–1060 26689474 10.1016/j.jfma.2015.10.010

[39] Choi JJE, Uy CE, Ramani RS, Waddell JN (2020) Evaluation of surface roughness, hardness, and elastic modulus of nanoparticle-containing light-polymerized denture glaze materials. J Mech Behav Biomed Mater 103:103601 32090930 10.1016/j.jmbbm.2019.103601

[40] Tekçe N, Fidan S, Tuncer S, Kara D, Demirci M (2018) The effect of glazing and aging on the surface properties of CAD/CAM resin blocks. J Adv Prosthodont 10(1):50–57 29503714 10.4047/jap.2018.10.1.50 PMC5829287

[41] Chen H, Wang R, Qian L, Ren Q, Jiang X, Zhu M (2018) Dental restorative resin composites: modification technologies for the matrix/filler interface. Macromol Mater Eng 303(10):1800264

[42] Lu H, Roeder LB, Lei L, Powers JM (2005) Effect of surface roughness on stain resistance of dental resin composites. J Esthet Restor Dent 17:102–108 16036126 10.1111/j.1708-8240.2005.tb00094.x

[43] Barutcigil Ç, Yildiz M (2012) Intrinsic and extrinsic discoloration of dimethacrylate and Silorane based composites. J Dent 40(1):57–63 22239912 10.1016/j.jdent.2011.12.017

